# Are acute psychiatric units providing adequate inpatient services for borderline personality disorder patients?

**DOI:** 10.1192/bjo.2021.867

**Published:** 2021-06-18

**Authors:** Siew Ling, Joji George

**Affiliations:** 1 University of Birmingham, Medical School; 2 National centre for Mental Health

## Abstract

**Aims:**

To assess the adherence to NICE guidelines CG78 (1.4) regarding the inpatient services provided for BPD patients at an acute psychiatric unit (The Oleaster).

Borderline personality disorder (BPD) patients are frequent users of psychiatric inpatient services. However, evidence suggests that inpatient treatment is ineffective in the long-term recovery of such patients. The inpatient services at the Oleaster will be audited against NICE guidelines for BPD. We hope to improve the care of patients with BPD and ensure effective use of psychiatric resources.

**Method:**

Retrospective case notes review of 35 patients admitted into the Oleaster from 1/11/2018–31/10/2019. This was taken from an initial sample of 72. Patients were excluded if they were admitted for other concomitant mental or behavioural problems (except problem use of tobacco, drugs or alcohol).

**Result:**

69% of patients were referred to other mental health services (e.g CRHT/HTT, other local alternatives, liaison team) prior to admission. There was no evidence of referrals in 31% of the sample population.

The reasons for admission include significant risks to themselves/others (n = 14) and detention under MHA (n = 14). Reasons were not noted in 7 patients.

Advance agreement on the length and purpose of admission took place in 19 and 27 patients respectively. Discussion of potential harms and benefits of admission only took place in 4 patients. Discussion was not applicable in 2 patients who lacked capacity.

Of the patients admitted ≥2 times in the previous 6 months, only 38% had a CPA review arranged. It was not arranged in the remaining 62%.

**Conclusion:**

There is room for improvement in the appropriate admission and documentation of BPD patients. Referral prior to admission was well adhered but documentation was unclear. Implementing a set checklist before admission could be recommended. Active involvement of patients was inadequate. It is especially lacking in regard to informing patients of the potential harms of admission. This can be improved by educating patients and staff on this matter.CPA reviews were not arranged in a timely manner. Placing an alert on patients’ records when they are admitted again within the last 6 months would help to reduce this issue. Overall, greater effort is required to ensure patient's most current needs are met and that limited psychiatric resources are used effectively.

